# Observations on Apoplexy

**Published:** 1802-07

**Authors:** 

**Affiliations:** Bath


### *To the Editors of the Medical and Phyfical Journal.*

GENTLEMEN,

THE difpute which has fome time exifted on the fubject of Apoplexy, a difeafe which, from its frequent occurrence, as well as too often fatal termination, fhould claim the attention of every confcientious practitioner, will, I doubt not, prove highly ferviceable to the caufe of medicine; for here, as in other branches of literature, it is only from oppofition of opinions and collifion of fentiment that truth can finally be elicited. Without oppofition the mind too frequently becomes torpid, and is unconfcious of its own powers; the validity of this affertion is proved in the falutary inducements to unreferved communication of fentiment, afforded by the weekly meetings of the Medical Societies in London during the winter feffion. The Conductors of the Medical and Phyfical Journal are fulfilling their duty to the public, by freely, and at all times, admitting controverfial papers, that tend to the elucidation of truth; nor can it be at all neceffary that the real name of the author fhould be affixed, as it is the doctrine and not the reputation of the writer, that is to be canvaffed by the public. Neither antient nor modern authorities fhould have weight in the balance, but as their principles will bear the teft of well-tried experience. The divifion of apoplexy into ferous and fanguineous has long held; whether it really marks the actual or proximate caufe of the difeafe in its two feparate ftates, would certainly admit of difpute, and perhaps at laft the point could not be afcertained, as it is probable that the effufion of blood or ferum may be an effect and not a caufe of the difeafes in queftion. In the fanguineous apoplexy we have every reafon, from the attendant fymptoms, to conclude that the preffure on the brain is from turgefcency of the veffels, the full labouring pulfe, the diftended countenance, florid, and fometimes approaching to purple, the ftertorous breathing, and the like, marking fuch a ftate of congeftion. The remedies which have fucceeded in this ftate of the complaint (as often as fuccefs could be expected) have been bleedings, in proportion to the ftrength of the patient, meafuring the quantity of blood to be taken away by the pulfe, which fhould be felt at the carotids as well as the wrift, by the fenfation of the heat or cold on the furface, by the frequency of breathing, and by any efforts the patient may poffibly make. Blood has been generally taken from the bend of the arm, the temporal artery, or from the jugular vein, and the laft would appear to be beft calculated to relieve; highly ftimulating glifters, after the lofs of blood, may be employed with advantage. The application application of warmth to the extremities, bliftering the fhorn head, from the principle held out by the venerable Cullen, and which experience in many analogous cafes, will be found to juftify, by their power to prevent hæmorrhagic tendency in the veffels: Thefe are the more obvious indications, the outlines as it may be termed, to be filled up by the fagacity and attention of the practitioner.

In the ferous Apoplexy, or nervous, as it has been termed by fome, or by others the inirritable apoplexy, that condition of the difeafe which appears to arife from torpid action in the brain, preventing a due return of blood, and in which partial congeftion may be fufpected; the method of treatment, particularly as it refpects bleeding, muft be fomewhat different, the quantity to be taken away lefs, and the local method to be preferred to general, as by leeches or cupping. The bowels are here alfo to be acted on, both by the mouth and by injections, blifters applied to the infide of the arms and legs, ammonia in full dofes, and active emetics; thefe laft, in difeafes of torpidity, appearing to give general action, or as it has been called, roufing the fyftem. Emetics feem next to blifters, to have the power of equalizing the circulation, of inducing a general and comfortable determination to the furface. In inebriety, a ftate very nearly approaching to apoplexy, Nature very frequently, and with feeming advantage, relieves herfelf by fpontaneous vomiting; hence, even in the true fanguineous apoplexy, after due evacuations, whenever there fhall be efforts of the ftomach to throw up, and we have reafon to fufpect offenfive matter, either from quantity or quality, that effect may be encouraged with advantage to the patient; for the ftomach, under thefe circumftances, does not poffefs the power of propelling its contents over the pylorus; nor could it indeed be effected with fufficient celerity to anfwer the defired end. This, gentlemen is, in brief, the view I am induced to take of apoplexy, drawn from experience, and this has been the rule of my practice; but which I fhall be ready to alter, whenever a more fuccefsful plan can be offered, the refult of actual trial; till which, I think I may juftly fay to the difputants, "his utere mecum."

